# Category Decoding of Visual Stimuli From Human Brain Activity Using a Bidirectional Recurrent Neural Network to Simulate Bidirectional Information Flows in Human Visual Cortices

**DOI:** 10.3389/fnins.2019.00692

**Published:** 2019-07-09

**Authors:** Kai Qiao, Jian Chen, Linyuan Wang, Chi Zhang, Lei Zeng, Li Tong, Bin Yan

**Affiliations:** PLA Strategic Support Force Information Engineering University, Zhengzhou, China

**Keywords:** brain decoding, functional magnetic resonance imaging, bidirectional recurrent neural network, bidirectional information flows, bottom-up manner, top-down manner

## Abstract

Recently, visual encoding and decoding based on functional magnetic resonance imaging (fMRI) has had many achievements with the rapid development of deep network computation. In the human vision system, when people process the perceived visual content, visual information flows from primary visual cortices to high-level visual cortices and also vice versa based on the bottom-up and top-down manners, respectively. Inspired by the bidirectional information flows, we proposed a bidirectional recurrent neural network (BRNN)-based method to decode the corresponding categories from fMRI data. The forward and backward directions in the BRNN module characterized the bottom-up and top-down manners, respectively. The proposed method regarded the selected voxels in each visual area (V1, V2, V3, V4, and LO) as one node of the space sequence and fed it into the BRNN module, then combined the output of the BRNN module to decode categories with the subsequent fully connected softmax layer. This new method can use the hierarchical information representations and bidirectional information flows in human visual cortices more efficiently. Experiments demonstrated that our method could improve the accuracy of the three-level category decoding. Comparative analysis validated and revealed that correlative representations of categories were included in visual cortices because of the bidirectional information flows, in addition to the hierarchical, distributed, and complementary representations that accorded with previous studies.

## Introduction

In neuroscience, visual decoding has been an important way to understand how and what sensory information is encoded and presented in visual cortices. Functional magnetic resonance imaging (fMRI) is an effective tool to reflect brain activities, and visual decoding computation models based on fMRI data have attracted considerable attention over the years (Kamitani and Tong, 2005; Haynes and Rees, 2006; Norman et al., 2006; Naselaris et al., 2011; Nishimoto et al., 2011; Horikawa et al., 2013; Li et al., 2018; Papadimitriou et al., 2018). Categorization, identification, and reconstruction of visual stimuli based on fMRI data are the three main means to visual decoding. Compared with identification and reconstruction, categorization or category decoding is common and feasible in the visual decoding domain, because identification is limited to fixed image dataset and fine reconstruction is limited to simple image stimuli.

The category decoding of visual stimuli can be mainly summarized into three kinds of methods: (1) classifier-based methods, (2) voxel pattern template matching-based methods, and (3) feature pattern template matching-based methods. Classifier-based methods accomplish category decoding by training a statistical linear or non-linear classifier to directly learn the mapping from specific voxel patterns in visual cortices to the categories. Previous work (Cox and Savoy, 2003) employed linear support vector machine (SVM) classifiers (Chang and Lin, 2011) to discriminate voxel patterns evoked by each category. In addition, various classifiers, including the Fisher classifier and k-nearest neighbor classifier have been also used (Misaki et al., 2010; Song et al., 2011). Wen et al. (2017) employed the classifier of the pre-trained convolutional neural network (CNN) (LeCun et al., 1998) to decode categories. Voxel pattern template matching-based methods need to compute the correlation between voxels to be decoded and the voxel pattern template of each category, and the category decoding can be accomplished according to the maximum correlation. The voxel pattern template for each category (Sorger et al., 2012) needs to be constructed in these methods. Haxby et al. (2001) directly used the means of the voxels of the samples with the same category as the voxel pattern template of each category. Kay et al. (2008) built an encoding model to predict the voxel patterns using those samples with a corresponding category and took the average of the predicted voxel patterns as the voxel pattern template of each category. Feature pattern template matching-based methods realize the decoding by mapping voxels to specific image features, comparing them to the feature pattern templates of each category and finally selecting the category with the maximum correlation. The third manner depends on the intermediate feature bridge, and the mapping from voxels to feature representations plays an important role. Horikawa and Kamitani (2017a) and Wen et al. (2018) constructed a feature pattern template for each category by averaging the predicted CNN features of all image stimuli belonging to the same category. Among these studies, the research based on hierarchical CNN features has received much attention (Agrawal et al., 2014; Güçlü and van Gerven, 2015).

In the human vision system, visual cortices are functionally organized into the ventral stream and the dorsal stream (Mishkin et al., 1983), and the ventral cortices are mainly responsible for object recognition. Anatomical studies have demonstrated that connections between the ventral cortices were ascending and also descending (Bar, 2003). The bidirectional (forward and backward) connections provide an anatomical structure for the bidirectional information flows in visual cortices. The forward (Tanaka, 1996) and backward information (Eger et al., 2006) flows play different roles in recognition tasks. Visual information flows from primary visual cortices to high-level visual cortices, and then we can obtain high-level semantic understanding, which is known as the bottom-up visual mechanism (Logothetis and Sheinberg, 1996). In this way, activities of visual cortices are mainly modulated by sensory input. Beside the forward inputs, the feedback modulation from high-level visual cortices can also affect the activities of low-level visual cortices (Buschman and Miller, 2007; Zhang et al., 2008). In this way, visual information flows from high-level visual cortices to low-level visual cortices, which is known as the top-down visual mechanism (Beck and Kastner, 2009; McMains and Kastner, 2011; Shea, 2015).

The top-down role in representations of visual cortices can be facilitated and enhanced under a task or goal (Beck and Kastner, 2009; Khan et al., 2009; Stokes et al., 2009; Gilbert and Li, 2013). For example, Li et al. (2004) demonstrated that neurons can carry more information about the stimulus attributes based on the top-down manner when people perform a task. Horikawa and Kamitani (2017a) showed that the categories of imaginary images can be decoded, and Senden et al. (2019) concluded that imagined letters can be reconstructed from early visual cortices, which revealed the tight correspondence between visual mental imagery and perception. These studies implied that visual information can flow from high-level visual cortices to modulate representations of low-level cortices based on the top-down manner. Moreover, for those without tasks or goals during recognition, visual attention (Kastner and Ungerleider, 2000; Baluch and Itti, 2011; Carrasco, 2011) seems also to be able to facilitate the top-down role in representations of visual cortices. People can choose to pay attention to the regions of interest on the basis of the visual attention mechanism after obtaining the semantic understanding of sensory input. In this way, semantic information can also flow from high-level visual cortices to modulate representations of the low-level visual cortices.

Although many works focused on the interactions (McMains and Kastner, 2011; Coco et al., 2014) between bottom-up and top-down manners, it is still unclear what is “top” and what is “bottom” in the debate about top-down influences on perception (Teufel and Nanay, 2017). However, the current anatomical and function roles of the bottom-up and top-down visual mechanism indeed indicate some important viewpoints. High-level visual cortices can form semantic representations or knowledge by hierarchical information processing based on the bottom-up manner, and representations in low-level visual cortices can also be modulated based on the top-down manner. In addition, a human subject viewed the same image stimulus in several repeated trials during the experiment of visual decoding, and the subject would pay attention to those interesting areas after grasping the main meaning of the image stimulus, because humans can only focus on one part at a time due to the visual bias competition (Desimone and Duncan, 1995). During the processing of visual information in a bottom-up and top-down manner, visual information frequently flows from low-level visual cortices to high-level visual cortices and the reverse direction. Thus, we can assume that the bidirectional information flows carry semantic knowledge from high-level visual cortices. Therefore, maximizing the bidirectional information flows in visual cortices will have great significant for category decoding.

However, the three types of category decoding methods ignored the internal relationship between different visual areas and regarded voxels in selected visual cortices as a whole to feed into the decoding model. Therefore, we introduced the bidirectional information flows into our decoding model to characterize the internal relationship. Compared to feedforward neural networks, recurrent neural networks (RNNs) (Mikolov et al., 2010; Graves et al., 2013; LeCun et al., 2015) can perform extremely well on temporal data and are widely used in sequence modeling. The general RNNs usually have only one directional connection from past to future (or from left to right) nodes of the input sequence. Bidirectional recurrent neural networks (BRNNs) (Schuster and Paliwal, 1997; Schmidhuber, 2015) split the neurons of regular RNNs into positive and negative directions. The two directions make it possible to use input information from the past and future of the current time frame. Inspired by BRNNs, we regarded the bidirectional information flows (one space sequence) as one fake temporal sequence. Therefore, we proposed to feed voxels in each visual area as one node of the sequence into the bidirectional connection module (Hochreiter and Schmidhuber, 1997; Sutskever et al., 2014). Thus, the output of the bidirectional RNN module can be regarded as the representations of the bottom-up and top-down manners. The category can be predicted with a subsequent fully connected softmax layer by combining the bidirectional representations.

In this study, our main contributions are as follows: (1) we analyzed the drawbacks of current decoding methods based on the bottom-up and top-down visual mechanisms, (2) we proposed to employ the BRNN to simulate the bidirectional information flows for the category decoding of visual stimuli, and (3) we analyzed that the bidirectional information flows make the internal relationship between visual areas related with the category, and validated that modeling the internal relationship was of significance for the category decoding.

## Materials and Methods

### Experimental Data

The dataset employed in our work was constructed based on the previous studies (Kay et al., 2008; Naselaris et al., 2009). The dataset had visual stimuli, corresponding fMRI data and category labels, consisting of 1750 training samples and 120 validating samples. The detailed information about the visual stimuli and fMRI data can be gained from previous studies (Kay et al., 2008; Naselaris et al., 2009), and the dataset can be downloaded from http://crcns.org/data-sets/vc/vim-1.

#### Visual Stimuli

The visual stimuli consisted of sequences of natural photographs, which were mainly obtained from the famous Berkeley Segmentation Dataset (Martin et al., 2001). The content of the photographs included animals, buildings, food, humans, indoor scenes, manmade objects, outdoor scenes, and textures. Photographs were converted into grayscale and downsampled to 500 pixels. The photographs (500 × 500 pixels) presented to subjects were obtained by centrally cropping, masking with a cycle, placing on a gray background, and adding a white square with size of 4 × 4 pixels in the central position. In total, 1870 images were selected as visual stimuli, and they were divided into 1750 and 120 images for training and validating, respectively.

#### Experiment Design

Photographs were presented in successive 4s trials. Each trial contained 1 s of presenting the photograph with a 200 ms flashing frequency and 3 s of presenting a gray presentation. The corresponding fMRI data was collected when two subjects with normal or corrected-to-normal vision viewed the photographs and focused on the central white square of the photographs. The experiment of each subject was composed of five scan sessions, and each session had five training runs and two validating runs. Seventy different images were presented two times during every training run and 12 different images were presented 13 times during the validating run. Images were randomly selected and were different for each run. Therefore, all 1750 different (5 sessions × 5 runs × 70) images and 120 different (5 sessions × 2 runs × 12) images for training and validating were presented to the subjects.

#### fMRI Data Collection and Pre-processing

The 4T INOVA MRI system with a quadrature transmit/receive surface coil was used to acquire fMRI data. Functional and anatomical brain volumes were reconstructed with the ReconTools software package https://github.com/matthew-brett/recon-tools. The repetition time (TR) was 1 s and isotropic voxel size was 2 × 2 × 2.5 mm^3^ in the single-shot gradient EPI sequence. The acquired data was subjected to a series of pre-processing, including phase correction, sinc interpolation, motion correction, and co-registration with the anatomical scan. Regarding the time-series of pre-processing for each voxel, voxel-specific response time courses were estimated based on the basis-restricted separable (BRS) model, and an estimate of the amplitude (a single value) of the voxel responses for each image was produced by deconvolving response time courses from the time-series data for repeated trials. The responses were then standardized to improve the consistency of responses across scan sessions. Voxels were assigned to each visual area based on the retinotopic mapping experiment performed in separate sessions. Voxels in five regions of interest (V1, V2, V3, V4, and LO) from low-level to high-level visual cortices were collected in the dataset.

#### Category Labels

**FIGURE 1:**
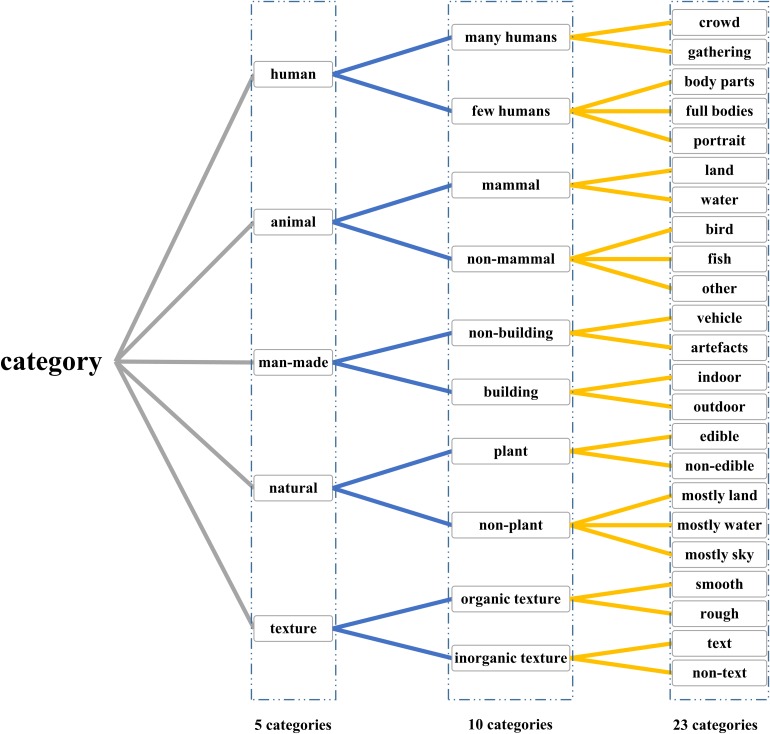
Three-level labels that have 5, 10, and 23 categories. Three-level categories were designed to validate the proposed method according to different grains, which can make the comparison more persuasive.

In addition to image stimuli and corresponding fMRI data, five experienced persons manually labeled the 1870 images, respectively, according to the three levels (5, 10, and 23 categories), and final labels were obtained through voting. As shown in Figure 1, the dataset with three-level categories can comprehensively validate the decoding method from coarse grains to fine grains.

#### Samples (Data Tuples) in Training and Validating

Each sample included one image stimulus, the corresponding preprocessed fMRI data, and three labels for three-level categories. Image stimulus was resized 224 × 224 to fit the input of the encoding model (see section “Visual Encoding Based on CNN Features”). It should be underlined that the fMRI data of samples does not have the dimension of time. The fMRI data removed the dimension of time through pre-processing, and each voxel in visual areas had one response value for one viewed image. One hundred voxels (one vector) in each visual area were selected based on the encoding model. Three labels in each sample were used for different levels of categorization. Because 1750 training images and 120 validating images were shown to two subjects, the dataset contained 1750 training samples and 120 validating samples for each subject.

### Overview of the Proposed Method

**FIGURE 2:**
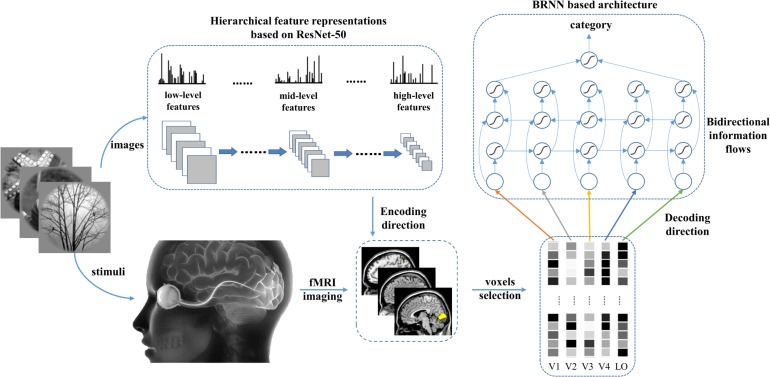
The proposed method. Hierarchical features in the deep network were used to predict voxel patterns in each visual area for the encoding direction. Based on the performance, the valuable voxels can be selected to reduce the dimension of voxels to a fixed number. To predict the category, the voxel sequence comprising five visual areas is fed into the BRNN-based method to extract semantic information from each visual area and the bidirectional information flows in visual cortices.

To introduce the bidirectional information flows into the decoding method, we employed a BRNN-based method to simulate the bottom-up and top-down manners in the human vision system. Thus, not only information of each visual area but also the internal relationship between visual cortices can be used in the decoding method. As shown in Figure 2, the proposed model included the encoding and decoding parts. For the encoding part, we can obtain the corresponding features of the given image stimuli based on the prevailing pre-trained ResNet-50 (He et al., 2016) model and employ these features to fit each voxel to construct the voxel-wise encoding model. According to the fitting performance, we can measure the importance of each voxel for all visual areas. We selected the fixed small number of voxels with higher predictive correlation for each visual area (V1, V2, V3, V4, and LO) to prevent the subsequent decoding from overfitting. For the decoding part, we constructed a RNN module and employed the selected voxels of each visual area as the five nodes of sequence input to utilize both hierarchical visual representations and bidirectional information flows in visual cortices. Finally, we combined the extracted features of the bidirectional RNN module as the input of the last fully connected softmax classifier layer to predict the category.

Section “Visual Encoding Based on CNN Features” introduces how to construct the visual encoding model based on hierarchical CNN features. Section “Category Decoding Based on BRNN Features” demonstrates how to use a BRNN to simulate the bidirectional information flows to decode the category.

### Visual Encoding Based on CNN Features

The brain can be looked as a system that non-linearly maps sensory input into brain activity. The linearizing encoding model (Naselaris et al., 2011) is validated and recognized in many studies. Therefore, we used the linear encoding model that consisted of non-linear mapping from image space to feature space and a linear mapping from feature space to brain activity space.

#### Non-linear Mapping From Image Space to Feature Space Based on Pre-trained ResNet-50 Model

Many works (Agrawal et al., 2014; Yamins et al., 2014; Güçlü and van Gerven, 2015) have indicated that hierarchical visual features extracted through the pre-trained CNN model demonstrated strong correlation with neural activities of visual cortices, and the visual encoding based on CNN features obtained better encoding performance than those hand-designed features such as Gabor features (Kay et al., 2008). In this study, we used the prevailing deep network ResNet-50 to extract hierarchical features for visual encoding. The pre-trained ResNet-50 can recognize 1000 types of natural images (Russakovsky et al., 2015) with state-of-the-art performance, which demonstrated that the network possessed rich and powerful feature representations.

In the ResNet-50 model, 50 hidden layers were stacked into a bottom-up hierarchy. Besides the first convolutional layer, four modules (16 residual blocks with each block mainly comprising 3 convolutional layers) and the last fully connected softmax layer were included in the network. Detailed network configuration can be seen in Table 1. Compared with the previous classic AlexNet (Krizhevsky et al., 2012) model, ResNet-50 was much deeper and contained more fine-grained hierarchical features, which is of benefit for the encoding. In order to reduce the computational cost, we only selected some representative features, including outputs of the last AvgPooling operation and 16 residual blocks for visual encoding. Thus, we extracted 17 kinds of features for each stimulus (1750 training images and 120 validating images) to learn the mapping from specific kinds of features to each voxel in each visual area (V1, V2, V3, V4, and LO). In the experiment, the pre-trained ResNet-50 model can be downloaded from https://download.pytorch.org/models/resnet50-19c8e357.pth, under the prevailing PyTorch deep network framework (Ketkar, 2017).

**TABLE 1:** Structure of the ResNet-50 model.

**Index**	**1**	**2**	**3**	**4**	**5**	**6**	**7**	**8**	**9**	**10**	**11**	**12**	**13**	**14**	**15**	**16**	**17**	**18**
**Module**	**-**	**1**			**2**				**3**						**4**			**-**
Name	Conv 1	Block 1	Block 2	Block 3	Block 1	Block 2	Block 3	Block 4	Block 1	Block 2	Block 3	Block 4	Block 5	Block 6	Block 1	Block 2	Block 3	Avgpool
Channel	64	64	256	256	256	512	512	512	512	1024	1024	1024	1024	1024	1024	2048	2048	2048
Feature size	112 × 112	56 × 56	56 × 56	56 × 56	56 × 56	28 × 28	28 × 28	28 × 28	28 × 28	14 × 14	14 × 14	14 × 14	14 × 14	14 × 14	14 × 14	7 × 7	7 × 7	1 × 1

*All modules, layer names, corresponding channels, and feature sizes are presented. The channels become larger and feature sizes become smaller because of the down-sampling.*

#### Linear Mapping From Feature Space to Activity Space Based on Sparse Regression

For each layer, a linear regression model maps the feature vector X to each voxel *y*, and it is defined as follows:

(1) y=X⁢w

where *y* is an *m*-by-1 matrix and X is an *m*-by-*n* matrix, where *m* is the number of training samples and *n* is the dimension of the feature vector. *w*, an *n*-by-1 matrix, is the weighting vector to be trained. Table 1 presents the dimension of each selected feature vector. The number of training samples *m* (∼2 K) is considerably less than the dimension of features *n* (∼100 K), which is an ill-posed problem. Thus, we assumed that each voxel can be characterized by a small number of features in the feature vector and regularized *w* was sparse to prevent overfitting when training the mapping from the high dimension of the feature vector to one voxel. On the basis of the above assumption, the major problem can be expressed as follows:

(2) $$\min_{w}w_{0}\;\text{subject to}\,X\,w=y$$

**FIGURE 3:**
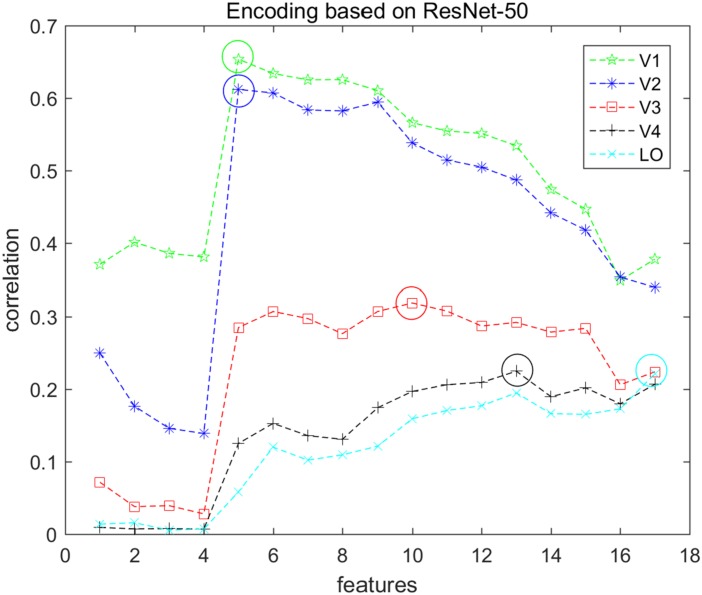
Encoding performance of each visual area based on ResNet-50 features. Seventeen types of features were used to encode each voxel in each visual area (V1, V2, V3, V4, and LO), and each node represents the average encoding performance of the top 200 voxels with higher correlation. Each color represents one type of visual area, and the corresponding “circle” indicates the optimal performance. In this way, the optimal features can be selected and the top 100 voxels were selected for each visual area.

**FIGURE 4:**
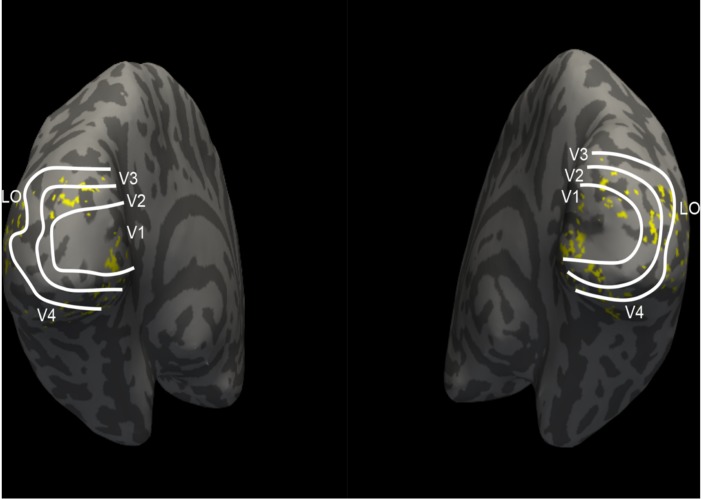
The distribution of selected voxels in visual areas. The white lines divide the five visual areas (V1, V2, V3, V4, and LO). Each yellow point represents one voxel, which indicates where 100 selected voxels of each visual area locate. These selected voxels are clustered instead of scattered distribution.

In this study, we employed a sparse optimization method called the regularized orthogonal matching pursuit (ROMP) (Needell and Vershynin, 2010) to fit the voxel pattern. ROMP adds an orthogonal item and group regularization based on the matching pursuit algorithm (Mallat and Zhang, 1993). Details of these algorithm steps can be found in Needell and Vershynin (2010). We constructed voxel-wise encoding models using each of the 17 different layers of features and optimized 17 linear models for each voxel. The correlation was used to measure the encoding performance, and the mean correlation of the top 200 voxels for each visual area was computed. The features that had the best correlation were selected as the final features for encoding that visual area. Figure 3 presents the encoding performance for each visual area when using a different layer of features. In the figure, the features of the optimal layer are marked in the “circle” according to the encoding performance. Finally, we selected the top 100 voxels for each visual area (V1, V2, V3, V4, and LO) according to the fitting performance, and a total of 500 voxels for five areas were selected for the next category decoding. On the basis of the encoding model, the dimension of voxels for each visual area was reduced to a small and fixed number, while valuable information was reserved. In addition, the encoding performance demonstrated that low-level features were better for encoding low-level visual cortices, and high-level features were appropriate for encoding high-level visual cortices, which was consistent with the previous study (Wen et al., 2018). Moreover, we illustrated that the selected voxels in visual areas shown in Figure 4 indicated the clustered distribution for selected voxels.

### Category Decoding Based on BRNN Features

In order to introduce bidirectional information flows to model the relationship between visual cortices, we used the prevailing long short-term memory (LSTM) module in the decoding method to extract the features about the category from the space sequence consisting of five visual areas. Then, the category could be predicted through fully connected softmax layer.

#### RNN Module

Long short-term memory (Hochreiter and Schmidhuber, 1997; Sutskever et al., 2014) is a famous RNN module in many RNN variants (Cho et al., 2014; Greff et al., 2016) and has been widely used in applications of sequence modeling. In this study, we employed bidirectional LSTM to characterize the bidirectional information flows in visual cortices, and bidirectional LSTM can be easily constructed by adding bidirectional (forward and backward) connections based on LSTM. Hence, we firstly overviewed the LSTM, and for detailed description the reader is referred to the following blog: http://colah.github.io/posts/2015-08-Understanding-LSTMs/.

Long short-term memory is normally augmented by recurrent gates called “forget” gates and can prevent backpropagated errors from vanishing or exploding. LSTM can learn tasks that require memories of events that occurred previously. LSTM includes three gates (“forget,” “input,” and “output” gates), which depend on previous state *h*_t–1_ and current input *x*_t_. The “forget” gate can control how much to forget previous information according to *f*_t_ computed through Equation (3), where σ represents the sigmoid function to restrict *f*_t_ from 0 to 1. In this way, LSTM can include long-term or short-term memory as needed by adjusting the *f*_t_. The “input” gate can control how much to feed current input *x*_t_ into the computation according to *i*_t_ computed through Equation (4). The “output” gate can control how much information to output according to *o*_t_ computed through Equation (5).

(3) $$f_{t}\,=\,\sigma \,\left(W_{f}\cdot \left[h_{t-1},x_{t}\right]+b_{f}\right)$$

(4) $$i_{t}\,=\,\sigma \,\left(W_{i}\cdot \left[h_{t-1},x_{t}\right]+b_{i}\right)$$

(5) $$o_{t}\,=\,\sigma \,\left(W_{o}\cdot \left[h_{t-1},x_{t}\right]+b_{o}\right)$$

On the basis of the three gates, LSTM can compute the state *c*_*t*_ and *h*_*t*_through the Equation (6) and (7), which is also the output of the current computation.

(6) $$c_{t}\,=\,f_{t}\cdot c_{t-1}+i_{t}\cdot \left\{\mathrm{𝑡𝑎𝑛ℎ}\,\left(W_{c}\cdot \left[h_{t-1},x_{t}\right]+b_{c}\right)\right\}$$

(7) $$h_{t}\,=\,o_{t}\cdot \mathrm{𝑡𝑎𝑛ℎ}\,\left(c_{t}\right)$$

#### The Proposed Architecture

The connections in the RNN module usually only have one direction (from left to right), but the BRNN adds the other direction (from right to left) to render the module bidirectional. Based on the bidirectional LSTM module, we presented the category decoding architecture.

**FIGURE 5:**
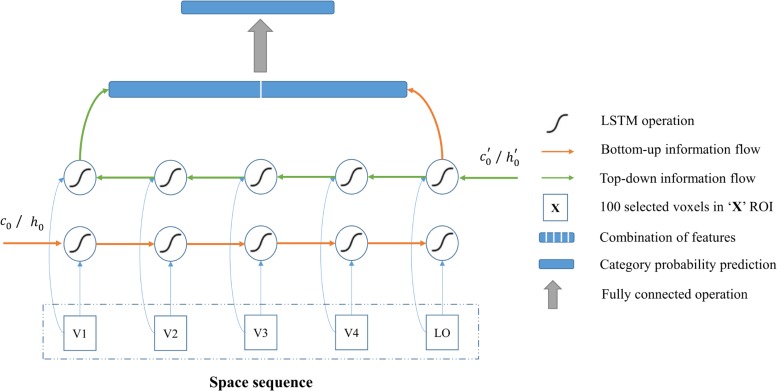
Category decoding model based on the BRNN module. All visual areas are regarded as one sequence, and the BRNN module is especially good at sequence modeling. The red line indicates the bottom-up information flows, and the green line indicates the top-down information flows in visual cortices. The combination of features from two directions are used to predict category. In this way, information from each visual area and bidirectional information flows in visual cortices can both be used for the decoding.

As shown in Figure 5, the input of architecture is the voxels selected from five visual areas (V1, V2, V3, V4 and LO), which comprise one space sequence, hence the length of the sequence is five. According to section “Visual Encoding Based on CNN Features,” we selected 100 voxels for each visual area. Because the voxels do not have the dimension of time, the 100 selected voxels from each area were regarded as one node (100-D vector) of the input sequence that was fed into the bidirectional LSTM module. In this way, each node can also be regarded as one moment (t_1_, t_2_, t_3_, t_4_, and t_5_) of the fake temporal input. Essentially, we employed the modeling of space sequence instead of time sequence for category, and we used bidirectional LSTM to characterize the space (several visual areas) series of the relationship instead of time series of the relationship for each voxel, which is the essential characteristic of our method.

One layer of bidirectional LSTM was added as the input layer in the decoding architecture to characterize the relationship in the input sequence. The directions from left to right and from right to left characterize the bottom-up and top-down manners in the human vision system, respectively. In this way, output features of one node are affected by the left low-level visual cortices and right high-level visual cortices. Hence, the features of category in each visual area and relationship between areas can be extracted. Then, the proposed method combined the output features from two directions and fed them into the successive fully connected softmax layer to predict the category. In addition, the focal loss (Lin et al., 2017) with the gamma 5.0 was used during the training to deal with the difficult samples. Regarding the details for the architecture, the input node was 100-D and the output of the node in each direction of LSTM was a 16-D feature. Thus, a 32-D feature combining two directions was obtained for the next classification. The number of nodes in the last fully connected softmax layer was 5, 10, and 23 for three-level labels, respectively. We added the dropout operation with a rate of 0.5 behind the output of bidirectional LSTM to avoid overfitting. Finally, not only visual information in each visual area but also the relationship between areas were used in the decoding model.

The proposed method can be trained in an end-to-end manner using similar algorithms as standard RNN. Through training under PyTorch deep network framework (Ketkar, 2017), the bidirectional information flows, including category information, can be mined on the basis of training samples. During the training, we set the batch size as 64 and used Adam optimization, in which the learning rate was 0.001 and the weight regularization was 0.001, to update parameters. About 200 epochs were required to accomplish the training on the Ubuntu 16.04 system with one NVIDIA Titan Xp graphics card.

## Results

### Conventional Linear and Non-linear Classifiers

**FIGURE 6:**
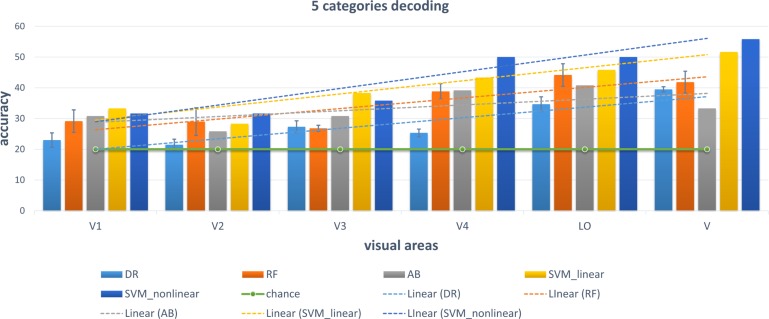
Decoding of five categories using conventional classifiers. Accuracies of different conventional classifiers when using only a single visual area and all visual areas (“V”) are presented. The distributed, hierarchical, and complementary representations of the semantic category in the human vision system can be observed (detailed analysis in the Discussion part).

**FIGURE 7:**
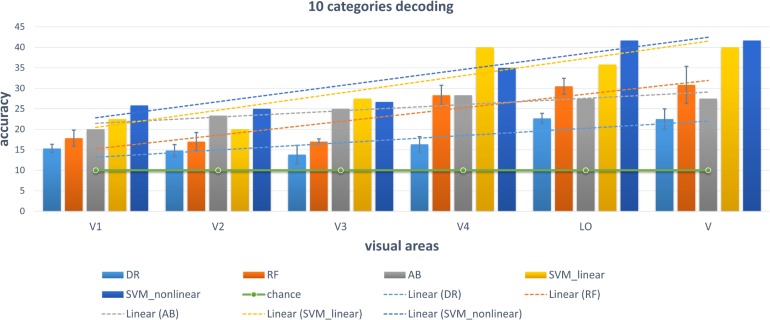
Decoding of 10 categories using conventional classifiers.

**FIGURE 8:**
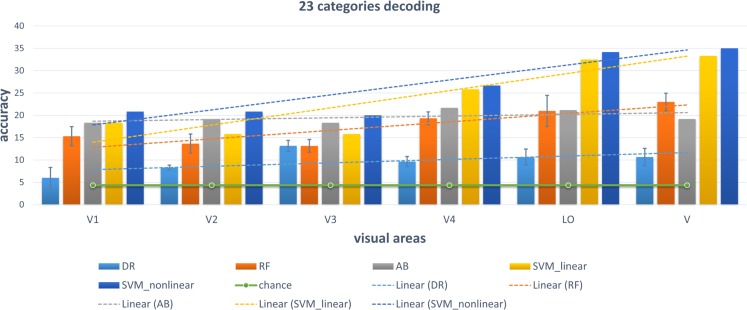
Decoding of 23 categories using conventional classifiers.

We chose some classical classifiers, including decision tree (DR), random forest (RF), AdaBoost (AB), linear and non-linear SVM. The three-level category (5, 10, and 23) decoding was performed on the basis of these conventional classifiers. For the 5-category decoding in Figure 6, these conventional methods using a single visual area were more accurate than random, and even primary visual regions are beneficial for semantic category decoding. The linear trend of decoding performance from low-level to high-level visual cortices is also depicted in the Figure, which shows that the decoding performance had been improved. This phenomenon indicated that more semantic information was obtained from higher-level visual areas. In addition, these classical classifiers obtained better decoding performance when all visual regions were used instead of a single visual region, which indicated that representations of category in different visual regions were complementary. The results of the other two levels (10 and 23 categories) of decoding demonstrated a similar phenomenon, which was shown in Figures 7, 8. Additionally, the mean and variance of decoding accuracy through five repeated experiment tests with the same hyper parameters were calculated and plotted in Figures 6–8. It should be noted that the variance of the stable linear and non-linear SVM and AB classifier was zero. We can see from the Figures that the decoding accuracy of SVM was higher than that of other methods (DR, RF, and AB) and the performance of the linear and non-linear SVM was similar. In addition, the performance of S1 was higher than that of S2, which accorded with previous studies (Kay et al., 2008).

### Fully Connected Neural Network

**FIGURE 9:**
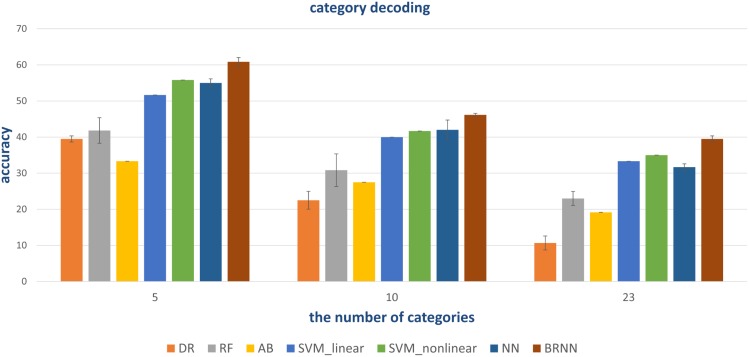
Quantitative comparison of decoding performance for different methods. Conventional methods and the NN method can employ all visual areas. However, the NN method with powerful non-linear capability obtains higher accuracy. BRNN-based methods with powerful non-linear capability can also employ additional information (bidirectional information flows), leading to the best performance.

In addition to the traditional classifiers in Section “Conventional Linear and Non-linear Classifiers,” the fully connected neural network (NN) method was also tested. In order to compare and validate the effect of modeling the bidirectional information flows, the NN method employed similar architecture as the proposed method except for the RNN module. In detail, the NN method had three fully connected layers. The number of neural nodes of each layer was 500, 64, and 32, respectively. The “500” was from the combination of selected voxels in five visual regions. The outputs of the last fully connected softmax layer were 5-D, 10-D, and 23-D for the three-level labels, respectively. Similar hyper parameters were employed during training. In this way, the difference between the NN- and BRNN-based methods was whether bidirectional information flows were modeled. From Figure 9, we can see that the NN method had better or comparative performance regarding the linear and non-linear SVM methods. We analyzed the gains benefited from the powerful non-linear ability of neural networks.

### The Proposed Method

**FIGURE 10:**
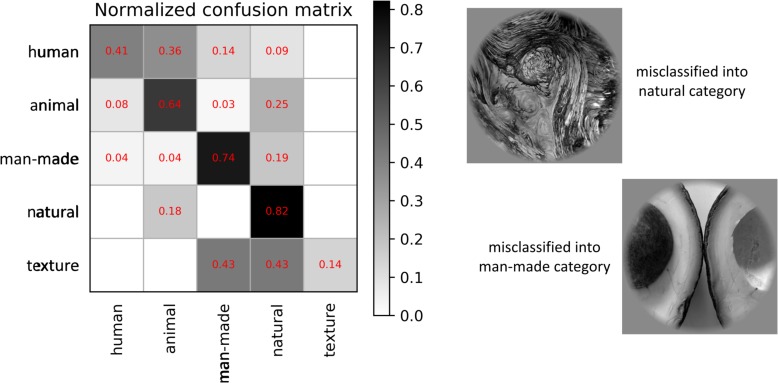
Normalized confusion matrix of results and two examples of the misclassification for the proposed method. The normalized confusion matrix presents detailed misclassification, and two image samples are used to analyze the class (“texture”) that has the worst classification performance.

As shown in Figure 9, our proposed method had the best performance for all three levels of category decoding because it can additionally utilize the bidirectional information flows in visual cortices. Table 2 gave the accuracy of our method, and the accuracy for 5-, 10- and 23-category decoding reached 60.83 ± 1.17%, 46.17 ± 0.42%, and 39.50 ± 0.85%, respectively. The proposed method obtained more than 5% improvement compared to the other best methods. Similar results for subject S2 can be found in Table 3. In order to validate the statistical significance, we calculated corresponding *p*-values to measure the difference between the proposed method and other classifiers in Table 4. It showed that most significance values reached the higher level (*P* < 0.001), which validated the significance of the proposed method. Moreover, the minimum significance values for each category level were underlined in Table 4, and the significance values were between (*P* < 0.01) and (*P* < 0.05), which demonstrated acceptable statistical significance. The underlined values indicated that our proposed method showed significance even though stricter comparisons were used, in which we compared the proposed method to the best of other all methods. In addition, Figure 10 presented the confusion matrix that reflected detailed classification results, and it was shown that the majority of samples were correctly classified. However, the class “texture” had the worst result, and we presented two images whose corresponding fMRI data were misclassified. One was misclassified into the class “natural,” and the other was misclassified into the class “man-made.” The visual attributes of the two images were indeed similar with those of images belonging to the “natural” and “man-made” classes. Moreover, the “human” and “animal” classes were easily confused, which may result from similar visual attributes between the “human” and “animal” classes.

**TABLE 2:** Quantitative comparison of decoding performance for different methods for subject S1.

**Category level**	**DR**	**RF**	**AB**	**Linear SVM**	**Non-linear SVM**	**NN**	**BRNN**
5	39.50 ± 0.85	41.83 ± 3.55	33.33	51.67	55.83	55.00 ± 1.13	60.83 ± 1.17
10	22.50 ± 2.47	30.83 ± 4.52	27.50	40.00	41.67	42.00 ± 2.72	46.17 ± 0.42
23	10.67 ± 1.93	23.00 ± 1.95	19.17	33.33	35.00	31.67 ± 0.91	39.50 ± 0.85

*The BRNN-based method obtains about 5% improvement than the other best method, which validates our proposed method and the significance of bidirectional information flows.*

**TABLE 3:** Quantitative comparison of decoding performance for different methods for subject S2.

**Category level**	**DR**	**RF**	**AB**	**Linear SVM**	**Non-linear SVM**	**NN**	**BRNN**
5	28.00 ± 0.85	30.83 ± 1.49	34.17	40.83	37.50	38.69 ± 1.69	42.50 ± 0.74
10	15.00 ± 2.64	22.00 ± 1.55	18.33	25.83	25.00	30.83 ± 1.39	36.33 ± 0.85
23	6.00 ± 0.63	14.50 ± 3.52	16.67	20.83	20.83	23.83 ± 2.15	26.33 ± 0.86

**TABLE 4:** Statistical significance of our proposed method compared to other methods for subject S1 and S2.

**Method**	**Category level**	**Linear SVM (S1/S2)**	**Non-linear SVM (S1/S2)**	**NN (S1/S2)**
	5	9.96 × 10^–5^/1.08 × 10^–2^^∗∗∗^/^*^	1.05 × 10^–3^/1.49 × 10^–5^^∗∗^/^∗∗∗^	5.47 × 10^–5^/7.46 × 10^–3^^∗∗∗^/^∗∗^
BRNN	10	7.38 × 10^–6^/1.59 × 10^–5^^∗∗∗^/^∗∗∗^	2.59 × 10^–5^/5.08 × 10^–7^^∗∗∗^/^∗∗∗^	3.66 × 10^–2^/3.40 × 10^–4^^*^/^∗∗∗^
	23	1.30 × 10^–4^/2.06 × 10^–4^^∗∗∗^/^∗∗∗^	4.49 × 10^–4^/1.82 × 10^–5^^∗∗∗^/^∗∗∗^	1.58 × 10^–6^/8.01 × 10^–2^^∗∗∗^/^*^

Differs between two methods: ^*^P < 0.05; ^∗∗^*P* < 0.01; ^∗∗∗^*P* < 0.001.

### The Effect of Feedforward, Backward and Bidirectional Connections

Furthermore, we compared the accuracy of the RNN module when using feedforward, backward and bidirectional connections. The bidirectional connections included the feedforward and backward connections. The feedforward connections characterized the bottom-up information flows, and the backward connections characterized the top-down information flows in visual areas. We compared the bidirectional connections (bidirectional LSTM) with forward connections (LSTM with the input of V1→ *V*2→ *V*3→ *V*4 →LO sequence), backward connections (LSTM with the input of LO→ *V*4→ *V*3→ V2→V1 sequence), and no recurrent connections (fully connected layer with the input of entire visual areas as whole). Corresponding results were presented in Table 5. We can see that the LSTM (“→→”)-based method that characterized the bottom-up information flows behaved better than the NN method without recurrent connections and the LSTM (“←←”)-based method that characterized the top-down information flows. However, there were still benefits after using bidirectional connections, because more relationships were characterized and more visual information was utilized. The bidirectional LSTM-based methods overall behaved the best according to the mean value in Table 5 through combining the LSTM (“→→”) and LSTM (“←←”) connections. We also computed the significance values to measure the difference between LSTM (“→→”) and bidirectional LSTM (“→→ and ←←”). For subject 1, the significance values for 5-, 10-, and 23-category decoding were 7.83 × 10^–4^, 7.72 × 10^–3^, and 4.34 × 10^–5^, respectively. For subject 2, the significance values for 5-, 10-, and 23-category decoding were 3.07 × 10^–1^, 2.41 × 10^–2^, and 5.31 × 10^–3^, respectively. These results showed the certain difference between LSTM (“→→”) and bidirectional LSTM (“→→ and ←←”), and the accuracies of the decoding task for subject 1 have higher significance values than for subject 2. In conclusion, the single LSTM (“←←”) connections behaved slightly worse than the NN-based method, but the improvement validated the role of the LSTM (“←←”). Therefore, bidirectional connections that characterized the bottom-up and top-down information flows are necessary for the decoding.

**TABLE 5:** The comparison about whether using feedforward, backward, and bidirectional connections for the RNN module.

**Category level**	**NN (S1/S2)**	**LSTM (→→) (S1/S2)**	**LSTM (←←) (S1/S2)**	**Bidirectional LSTM (→→ ←←) (S1/S2)**
5	55.00 ± 1.13/ 38.69 ± 1.69	56.83 ± 0.97/ 41.83 ± 0.95	49.17 ± 0.91/ 39.83 ± 1.62	60.83 ± 1.17/ 42.50 ± 0.74
10	42.00 ± 2.72/ 30.83 ± 1.39	44.50 ± 0.85/ 34.67 ± 0.57	39.73 ± 1.23/ 30.17 ± 0.63	46.17 ± 0.42/ 36.33 ± 0.85
23	31.67 ± 0.91/ 23.83 ± 2.15	34.33 ± 0.97/ 24.33 ± 0.62	31.50 ± 0.62/ 22.33 ± 0.97	39.50 ± 0.85/ 26.33 ± 0.86
**mean**	**37.00 ± 1.67**	**39.42 ± 0.82**	**35.46 ± 0.99**	**41.94 ± 0.96**

*“**→→**” represents the feedforward connections that characterize the input of V1**→**V2**→**V3**→**V4**→**LO sequence with the LSTM, “**←←**” represents the backward connections that characterize the input of the LO**→**V4**→**V3**→**V2**→**V1 sequence with the LSTM. BRNN module can characterize the bidirectional information flows including V1**→**V2**→**V3**→**V4**→**LO sequence and LO**→**V4**→**V3**→**V2**→**V1 sequence with bidirectional LSTM, and NN employed a plain fully connected neural network without recurrent connections. The “mean” represents the mean performance through averaging the three category levels and two subjects for each method.*

## Discussion

It is known that visual decoding is to explore what exists in visual cortices, but it is easier to explore the pattern of visual representations in the human vision system. Hence, we concluded some existing points and summarized the similarities and differences between our method and others. In addition, the CNN- and RNN-based methods for visual decoding were discussed to demonstrate the advantage and limitations of our proposed method, and our contribution to this domain was presented.

### Some Accordance With Previous Studies

It is known that low-level and high-level features of deep networks are focused on detailed and abstract information, respectively (Mahendran and Vedaldi, 2014). From the viewpoint of visual encoding, Figure 3 shows that the low-level and high-level features are suitable to encoding low-level and high-level visual cortices, respectively, which has been shown in a series of previous studies (Güçlü and van Gerven, 2015; Eickenberg et al., 2016; Horikawa and Kamitani, 2017b). From the viewpoint of visual decoding, Figures 6–8 of our study show a linear improvement from low-level visual cortices to high-level visual cortices, which can be a supplement to CNN-based visual encoding methods to support the hierarchical representations in visual cortices.

When only one specific visual area is used in different classifiers, the category decoding performance is better than random, and even the low-level visual areas can contribute to category decoding, which indicates that low-level visual areas can contain visual information of categories. Thus, just like the previous work (Haxby et al., 2001; Cox and Savoy, 2003), the distributed representations of categories in visual cortices can be concluded. For example, Haxby et al. (2001) demonstrated that there were distributed representations of faces and objects in ventral cortices. Based on the bidirectional information flows, we suggested that the distributed representations may be caused by the dynamic information flows. The visual information of low-level visual areas can flow to high-level visual areas, and visual information of high-level visual cortical areas can also flow to low-level visual cortical areas. Therefore, the visual areas in ventral cortices are interactive, which may make the representations in visual cortices distributed.

The results reveal that the decoding performance using five visual areas is superior to using only one single area. The improvement validates that these representations in different visual areas are not redundant but contain various information. The encoding results based on hierarchical CNN (see Figure 3) have revealed that low-level features are suitable for encoding primary visual cortices, and high-level features are more useful for encoding high-level visual cortices. Considering that the low-level and high-level features of the deep network focused on detailed and abstract information (Mahendran and Vedaldi, 2014), the improvement supplements the viewpoint that low-level visual cortices mainly process low-level representations (edge, texture, and color) and that high-level visual cortices are mainly responsible for high-level representations (shape and object). Moreover, the complementary representations indicate that more visual areas should be considered. However, this study only covers five visual areas, which is a limitation, and some previous studies even mention fewer visual cortical regions (Senden et al., 2019). Hence, it might be the next direction to use more visual areas in the decoding method and to model more complex relationships in visual areas.

### Correlative Representations About the Category in Visual Cortices

Except hierarchical, distributed and complementary representations about categories in visual cortices, the results in Figure 9 demonstrated that we can obtain about 5% improvement after introducing the bidirectional information flows and modeling the internal relationship in the decoding method, which indicates that the relationship between visual areas may contain semantic information of categories and can contribute to the decoding. This shows that these visual areas are related, and the category representations in visual cortices are correlative. The correlative representations of categories mean that the relationship between visual areas contains the attributes about categories. Since we had not found literatures that modeled the correlative representations to decode categories from fMRI data, we tried to analyze the origin of the phenomenon according to the bidirectional information flows. Namely, semantic knowledge is firstly formed through bottom-up hierarchical processing of sensory input. Then, semantic information can flow from high-level visual cortices to modulate neural activities in low-level visual cortices because of the task or attention. Thus, we can conclude that the semantic information contained in the relationship derives from bottom-up visual processing and top-down visual modulating, and the relationship is related with categories due to the effect of a top-down manner. Current methods, such as prevailing CNN-based methods, fail in simulating the top-down visual mechanism and usually only consider the hierarchical representations.

### Difference From Prevailing Visual Decoding Method-Based CNNs and RNNs

The goal of our study is to directly decode categories from voxels (fMRI activities) using a classifier based on the RNN module. It has been known that CNNs are very efficient for visual recognition tasks through extracting hierarchical and powerful features from 2D images. Thus, CNNs are especially suitable for visual encoding but not for classifying voxels (1D). As shown in Section “Visual Encoding Based on CNN Features,” features extracted by the CNN are used to encode voxels to select valuable voxels. In addition, CNNs can perform decoding through an indirect manner called “Feature pattern template matching-based methods” (Han et al., 2017; Horikawa and Kamitani, 2017a), which is essentially different from our method called “Classifier-based methods,” which is the most direct way to decode. Besides, the kind of “Feature pattern template matching-based methods” takes entire visual areas as a whole and maps it to CNN features, which makes it difficult to exploit the inner relationship between voxels. However, RNNs are usually used to model the sequence data, and RNN-based methods (Spampinato et al., 2017; Shi et al., 2018) can characterize the data with the dimension of time in visual decoding domain. For example, Spampinato et al. (2017) proposed to employ the RNN to extract features from EEG data for decoding, and they used the LSTM module to characterize the time series of the relationship. As an improvement, we used the LSTM module to characterize the space (several visual areas) series of the relationship since the dimension of time for fMRI data usually is not considered too much in the visual encoding and decoding domain. More specifically, their sequence is composed of different time points for each voxel, but the sequence for our RNNs is composed of voxels in different visual areas, which is the essential difference between our method and other RNN-based methods. In conclusion, our method is direct and novel because we employ the modeling of space sequence instead of time sequence for category. Thus, the next direction for visual decoding might be to characterize the space-time sequence of voxels in visual areas.

## Conclusion

In this study, we analyzed the drawbacks of current decoding methods from the perspective of the bidirectional information flows (bottom-up and top-down visual mechanisms). In order to characterize the bidirectional information flows in visual cortices, we employed the BRNN module to model the space series of the relationship instead of the common time series of the relationship. We regarded the selected voxels of each visual area (V1, V2, V3, V4, and LO) as one node in the space sequence, which fed into the BRNN to additionally extract the relationship features related with category to improve decoding performance. We validated our proposed method on the dataset with three levels of (5, 10, and 23) category labels. Experimental results demonstrated that our proposed method was capable of more accurate decoding results than other linear and non-linear classifiers, while validating the statistical significance of bidirectional information flows for category decoding. In addition, based on experimental results, we concluded that representations in visual cortices were hierarchical, distributed, and complementary, which accorded with previous studies. More importantly, we analyzed that the bidirectional information flows in visual cortices made the relationship between areas contain representations of categories and can be successfully used based on BRNN, which we called correlative representations of categories in visual cortices.

## Author Contributions

KQ contributed to all stages of the research project and writing. JC designed the procedures of overall experiments. LW contributed to the idea of decoding based on the BRN. CZ contributed to the implementation of the idea. LZ contributed to the preparation of the article, figures, and charts. LT introduced the perception of hierarchical, distributed, complementary, and correlative representations in visual cortices. BY proposed the idea and writing.

## Conflict of Interest Statement

The authors declare that the research was conducted in the absence of any commercial or financial relationships that could be construed as a potential conflict of interest.
